# Serum zinc levels in seborrheic dermatitis: a case-control study

**DOI:** 10.3906/sag-1906-72

**Published:** 2019-10-24

**Authors:** Ezgi AKTAŞ KARABAY, Aslı AKSU ÇERMAN

**Affiliations:** 1 Department of Dermatology and Venereology, Faculty of Medicine, Bahçeşehir University, İstanbul Turkey; 2 Department of Dermatology and Venereology, Health Sciences University, Şişli Hamidiye Etfal Training and Research Hospital, İstanbul Turkey

**Keywords:** Immune system, inflammation, seborrheic dermatitis, zinc

## Abstract

**Background/Aim:**

Malassezia colonization, sebaceous gland activity, hormones, immune system defects, environmental factors, and the interactions between these factors are thought to contribute to the pathogenesis of seborrheic dermatitis (SD). Zinc, an essential element, is involved in many biological processes including the ones that contribute to the development of SD. The aim of this study is to evaluate serum zinc levels in patients with SD.

**Materials and methods:**

Forty-three patients with SD and 41 healthy controls were enrolled in the study. Disease activity was assessed by the Seborrheic Dermatitis Area and Severity Index by a single dermatologist. Serum zinc levels of all subjects were evaluated.

**Results:**

Statistically significantly lower serum zinc levels were noted in SD patients than in the control group (79.16 ± 12.17 vs. 84.88 ± 13.59, respectively; P = 0.045).

**Conclusion:**

The results of the study demonstrated that patients who had SD had lower levels of serum zinc levels than healthy subjects.

## 1. Introduction

Seborrheic dermatitis (SD) is a chronic inflammatory skin disease localized to areas rich in sebaceous glands, such as the scalp, face, upper chest, and back [1]. Various factors contribute to the pathogenesis of SD, including hormonal factors, comorbidities (associated diseases), individual immunological features, inflammatory status, and nutritional, environmental, and lifestyle factors, but the exact etiology of the disease has not been clarified [2].

Zinc is a mineral involved in many biological processes, including immune functions and metabolic and hormonal pathways. It may play a role in the different steps of the cutaneous inflammatory reactions, inhibiting the chemotaxis of neutrophils, activating natural killer (NK) cells, and modulating the production of proinflammatory cytokines. In addition, zinc displays antioxidant and antiandrogen activity [3]. Zinc is considered a contributor in the pathogenesis of several inflammatory skin diseases associated with innate immunity dysregulation, such as inflammatory acne, folliculitis decalvans, and hidradenitis suppurativa (HS) [4]. It has been reported that patients with severe acne and HS have lower serum zinc levels than the healthy population [5–8]. Moreover, SD-like dermatitis has also been reported to be associated with zinc deficiency [9,10]. Among many functions, zinc also plays a role in some of the biological processes that contribute to the development of SD. However, no reports are available investigating serum zinc levels in patients with SD.

The aim of this study was to determine the association between SD and serum zinc levels.

## 2. Materials and methods

The study was reviewed and approved by the local ethics committee (protocol number: 22481095-020-1958, date of approval: 19/09/2018), and all individuals gave written informed consent. The study was carried out according to the principles expressed in the Declaration of Helsinki.

A prospective case-control study was designed to investigate the relationship between serum zinc levels and SD. Forty-three patients diagnosed with SD by clinical or histopathological examination were recruited from a dermatology outpatient clinic. For comparison, 41 healthy age- and sex-matched controls with no evidence of SD were recruited from among hospital staff volunteers. Only those with a normal body mass index (BMI) (18.5– 25 kg/m2) were included. Subjects taking zinc salts or multivitamins containing zinc, or under any systemic treatment, including corticosteroids, retinoids, antifungal agents, and immunosuppressants within 6 months of the study, were excluded. Subjects with a history of any disease or condition that can present with serum zinc level alterations, such as inflammatory acne, folliculitis decalvans, enteropathic acrodermatitis, malabsorptive diseases, malnutrition, strict diet, or high alcohol consumption (more than 20 g/day for women and more than 30 g/day for men) [11], were also excluded. Subjects with any inflammatory conditions that may be associated with immune disruption, such as inflammatory bowel disease, rheumatoid arthritis, ankylosing spondylitis, psoriasis, and any other systemic diseases (e.g., diabetes mellitus, parathyroid or thyroid disorders, autoimmune diseases, anemia, atopy, chronic renal or liver disease, and malignancy), as well as currently pregnant or lactating females and smokers, were also excluded. The data on smoking relied on self-reports.

The data on baseline demographics, clinical characteristics, and blood test results were obtained on the same day. Serum zinc levels were measured in all subjects using fasting venous blood samples. Venous blood samples were drawn from the participants between the hours of 09:00 and 11:00 AM following a 12-h fasting period. The measurements of serum zinc levels were taken with an atomic absorption spectrophotometric system (PerkinElmer, Norwalk, CT, USA). Normal values were defined as those above 60 μg/dL and below 120 μg/dL.

SD was graded according to the SD Area and Severity Index (SDASI), which was modified from the Psoriasis Area Severity Index (PASI) established by Cömert et al. [12]. The SDASI was calculated at the time of blood collection by a single dermatologist. According to this scoring system, the erythema and desquamation of nine different anatomical sites were graded on a scale between 0 and 3, where 0 = none, 1 = mild, 2 = moderate, and 3 = severe. The score of each site was multiplied by the constant for the area (forehead [0.1], scalp [0.4], nasolabial [0.1], eyebrow [0.1], postauricular [0.1], auricular [0.1], intermammary [0.2], back [0.2], and cheek or chin [0.1]), and the sum was determined as the SDASI score (range: 0–12.6) [12].

### 2.1. Statistical analysis

The Number Cruncher Statistical System 2007 program (NCSS; Kaysville, UT, USA) was used for statistical analysis. Descriptive data were expressed with mean ± standard deviation, numeric variables, and percentages. In the analysis of normally distributed variables, the Shapiro– Wilk test and graphical analysis were applied to examine the differences between the two groups. In the analysis of normally distributed variables, an independent samples t-test was applied to examine the differences between the two groups. The Pearson chi-square test was used to compare categorical variables. Correlation analysis was performed by calculating Pearson and Spearman rank correlations. Diagnosis and treatment tests (sensitivity, specificity, positive predictive value, and negative predictive value) and ROC analysis were used to define predictive values. P < 0.05 was considered statistically significant.

## 3. Results

A total of 43 patients with SD and 41 healthy control subjects were included in the study. No significant differences were observed in the sex ratio or ages between the patients with SD and healthy controls (P > 0.05). The mean disease duration among the SD patients was 17.49 ± 21.49 months, ranging from 1 to 120 months. The SDASI scores of the patients ranged from 0.8 to 6.6, with a mean of 2.79 ± 1.26.

Statistically significantly lower serum zinc levels were noted in SD patients than in the control group (79.16 ± 12.17 vs. 84.88 ± 13.59, respectively; P = 0.045). The clinical characteristics of the study group are shown in Table 1.

**Table 1:** Demographic data of the subjects.

			Group		Test value
		Total	SD patients (n = 43)	Control (n = 41)	P
Age (years)	Min–max (median)	19−53 (29.5)	20−44 (28)	19−53 (31)	t: –1.703
	Mean ± SD	30.60 ± 6.37	29.44 ± 4,90	31.80 ± 7.49	a0.093
Sex	Female	38 (45.2)	19 (44.2)	19 (46.3)	χ2: 0.039
	Male	46 (54.8)	24 (55.8)	22 (53.7)	b0.843
Serum zinc levels (μg/dL)	Min–max (median)	50−126 (81.5)	50−105 (77)	63−126 (82)	t: –2.033
	Meant ± SD	81.95 ± 13.12	79.16 ± 12.17	84.88 ± 13.59	a0.045*

aStudent t-test, bPearson chi-square test, *P <0.05. SD: Seborrheic dermatitis

In ROC curve analysis, the cutoff value for serum zinc levels was assessed as 79 μg/dL with a sensitivity of 58.14% and a specificity of 73.17%. The positive predictive value was 69.4 and the negative predictive value was 62.5 (Table 2; Figure). No correlations were found between serum zinc levels and disease duration (P = 0.658) or SDASI scores (P = 0.273) (Table 3).

**Table 2:** Diagnostic scan and ROC curve results of serum zinc levels in seborrheic dermatitis.

	Diagnostic scan					ROC curve		P
	Cut off	Sensitivity	Specificity	Positive predictive value	Negative predictive value	Area	95% confidence interval	
Serum zinc levels (μg/dL)	≤79	58.14	73.17	69.4	62.5	0.623	0.502−0.744	0.044*

*P < 0.05.

**Figure:**
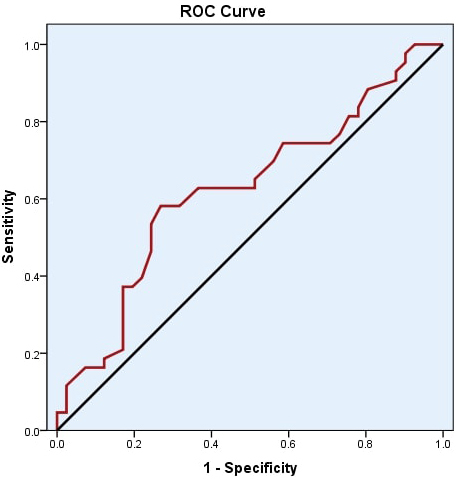
ROC curve analysis of serum zinc levels in SD

**Table 3:** Relationship between serum zinc levels and disease duration and SDASI.

		Serum zinc levels
Disase duration (months)	r	0.070c
	P	0.658
SDASI	r	-0.171d
	P	0.273

cr = Spearman rank correlation, dr = Pearson rank correlation. SDASI: Seborrheic Dermatitis Area and Severity Index.

## 4. Discussion

SD is a chronic inflammatory disease of the skin characterized by erythematous, oily, yellow squames that are located on sebum-rich areas, such as the face or the forehead [13]. Although the exact etiology of the disease is unknown, increased sebum activity, Malassezia infection, immunological abnormalities, androgens, emotional stress, diet, lifestyle, and environmental factors are thought to contribute to the pathogenesis of the disease [14]. The male dominancy of SD and the development of SD during puberty may indicate a significant effect of hormones, namely androgens, in the pathogenesis of the disease [15].

Sebaceous gland activity is thought to be correlated with SD development. The level of sebum production and abnormalities of lipid composition are thought to play a role in SD development and also provide a suitable environment for Malassezia growth [16].

Malassezia, a fungal component of normal human skin, is thought to play a role in the pathogenesis of SD [17]. Since it is a lipid-dependent microorganism, it is found on sebum-rich areas of the skin, similar to the involvement sites of SD [18]. Malassezia hydrolyzes sebum triglycerides into unsaturated fatty acids, such as oleic acid and arachidonic acid, by its lipase [19]. The metabolites of the yeasts induce inflammation with the infiltration of NK cells and macrophages and an increased local production of inflammatory cytokines such as interleukin (IL)-1α, IL- 1β, IL-6, and tumor necrosis factor (TNF)-α in lesional skin areas. These metabolites stimulate keratinocyte differentiation, leading to abnormalities in the stratum corneum that result in disruptions of the epidermal barrier function and inflammatory response [20,21].

Some evidence suggests that impairment in the epidermal barrier function due to altered corneodesmosomal hydrolysis, lipid disorganization, and abnormalities in the desquamation process also contribute to the pathogenesis of SD [20,21].

SD has been reported to be more common in immunosuppressed patients, particularly those with HIV/ AIDS [13]. The immune or inflammatory individual response to Malassezia was also considered a contributory factor [16,22]. Moreover, the levels of human leukocyte antigens (HLAs), including HLA-AW30, HLA-AW31, HLA-A32, HLA-B12, and HLA-B18, were reported to be elevated in SD patients [22], in addition to reports of increased levels of total serum IgA and IgG antibodies [23], suggesting the potential immune mechanisms involved in the pathogenesis of the disease.

The genetic components of SD have been studied in animal models and humans [13]. Zinc finger 750 (ZNF7509) is a transcription factor controlling epidermal differentiation and an upstream regulator of MPZL3. Autosomal dominantly inherited SD-like dermatitis has been identified in a frameshift mutation in ZNF750 [24]. The functional pathway of ZNF750-MPZL3 has been suggested to play an important role in the pathogenesis of SD [25].

Nutritional deficiencies, particularly of riboflavin, pyridoxine, niacin, and zinc, can also present as SD-like dermatitis by an unknown mechanism [9,10]. Although the exact pathogenesis is still unclarified on the basis of several studies, SD is considered a multifactorial disease, with immune, inflammatory, and environmental factors contributing.

Zinc is an essential element for the proper functioning of several processes in the human body. Among these, zinc plays a role in a number of skin disorders [26]. In both acquired and inherited forms of hypozincemia, cutaneous findings, including periorificial and acral dermatitis, alopecia, diaper rash, photosensitivity, nail dystrophy, angular stomatitis, angular cheilitis, eczematous annular plaques in areas of friction and pressure, dystrophic nails, structural hair changes, and diminished growth of both hair and nails, have been reported [27,28]. Zinc deficiency is reported in some inflammatory skin disorders, including atopic dermatitis [29], oral lichen planus [30], and Behçet’s disease [31], and in autoimmune bullous diseases such as pemphigus vulgaris [32], bullous pemphigoid [33], epidermolysis bullosa [34], and melasma [35]. It is thought that zinc plays a role in the development of these disorders via its effects on the immune system [36]. Also, lower serum zinc levels have been shown to be associated with the occurrence of acne vulgaris [6,37]. In a recent review, zinc was reported to be effective in the treatment of acne vulgaris [26].

Among various functions, zinc plays a role in many processes that may affect the development of SD [6,38–43]. Zinc affects the regulation of protein, lipid, and nucleic acid metabolism, acting as a cofactor in metalloenzymes and transcription factors. Zinc also plays a role in gene transcription via a zinc-finger motif containing proteins and factors. It also regulates cell replication, immune activity, and wound repair. Zinc provides proper immune activity by preserving macrophage and neutrophil function and by stimulating NK cell and complement activity. Zinc also has antiinflammatory effects through the inhibition of IL-6, TNF-α, nitric oxide, and integrin and toll-like receptor expression by keratinocyte production [6,41]. Additionally, the zinc finger-transactivating protein A20 inhibits IL-1b and tumor necrosis factor-α activation of nuclear factor (NF)-kB [43]. Moreover, zinc has antiandrogenic activity through the inhibition of 5α-reductase, which is the enzyme responsible for the conversion of testosterone to dihydrotestosterone. This also results in the suppression of sebaceous activity [6,43]. All the biologic processes mentioned above also occur in the development of SD [9–20]. We believe that zinc deficiency may play a role in the pathogenesis of the disease through various mechanisms. Additionally, topical zinc combinations have been reported to be effective in the treatment of SD [44]. Pierard et al. [44] reported that topical zinc formulation may be effective in the treatment of SD through the modulation of epithelial differentiation, antiinflammatory and antibacterial activity, and the inhibition of 5α-reductase, which provides antiandrogen activity [44].

Since most of the mechanisms involved in the development of SD are related to the functions of zinc, we hypothesized that SD patients may have zinc deficiency or lower zinc levels. The results of the study demonstrated that patients who had SD also had lower levels of serum zinc levels compared with healthy subjects. However, in the present study, serum zinc levels did not show any correlation with disease severity, which was presented as SDASI scores. We believe that there are two reasons for that: the sample size was small, and the patients included in the study had mild SD symptoms. It is known that 12.6 is the highest SDASI score that can be measured [12]; the highest SDASI score assessed in this study group was 6.6, while the mean score was 2.79 ± 1.26, which might be considered a very mild presentation of SD.

The patients enrolled in the study had mild or moderate forms of SD, which could be considered a limitation of the study. In studies conducted with patients with higher SDASI scores, the results might vary dramatically, perhaps demonstrating zinc deficiency. Small sample size is another limitation of the study.

In conclusion, zinc has many properties that affect inflammatory processes, the immune system, and epithelial differentiation, and it has antifungal properties and antiandrogenic effects, all of which also contribute to the pathogenesis of SD. Based on these data and the reports of SD-like dermatitis development in zincdeficient individuals, we hypothesized that SD patients might have lower serum zinc levels than those without the disease. To date, no data are available on serum zinc levels in SD. The present study revealed lower zinc levels in SD patients compared with controls. Further research on the association of zinc levels and SD will help identify the pathogenesis of the disease and help develop more efficacious disease management.

## References

[ref1] (2010). Seborrheic dermatitis. Pharmacology & Therapeutics.

[ref2] (2014). Topical anti-inflammatory agents for seborrhoeic
dermatitis of the face or scalp.. Cochrane Database of Systematic Reviews.

[ref3] (2007). Hidradenitis suppurativa and zinc: a new therapeutic approach. A pilot study.. Dermatology.

[ref4] (2011). Innate immunity: a crucial target for zinc
in the treatment of inflammatory dermatosis.. Journal of the
European Academy of Dermatology and Venereology.

[ref5] (2010). Innovative uses
for zinc in dermatology. Dermatologic Clinics.

[ref6] (2014). Evaluation of serum vitamins A and E and zinc levels according the severity of acne vulgaris. Cutaneous and Ocular Toxicology.

[ref7] (1982). Serum zinc in acne vulgaris. International Journal of Dermatology.

[ref8] (2018). Serum zinc levels in hidradenitis suppurativa: a casecontrol
study. American Journal of Clinical Dermatology.

[ref9] (2012). Seborrheic dermatitis: an update. Acta Dermatovenerologica Croatica.

[ref10] (2006). Etiopathogenesis of seborrheic dermatitis. Indian Journal of Dermatology, Venereology and Leprology.

[ref11] (2018). The bidirectional impacts of
alcohol consumption and the metabolic syndrome: cofactors
for progressive fatty liver disease.. Journal of Hepatology.

[ref12] (2010). Psychiatric
comorbidities and alexithymia in patients with seborrheic
dermatitis: a questionnaire study in Turkey.. American Journal
of Clinical Dermatology.

[ref13] (2015). Seborrheic dermatitis and dandruff: a comprehensive review. Journal of Clinical and Investigative Dermatology.

[ref14] (2004). Seborrheic dermatitis. Journal of the European Academy of Dermatology and Venereology.

[ref15] (2009). Clinical practice. Seborrheic dermatitis. New England Journal of Medicine.

[ref16] (1996). Skin surface lipids in HIV-positive patients with and without
seborrheic dermatitis. International Journal of Dermatology.

[ref17] (2011). Malassezia, dandruff and seborrhoeic dermatitis: an
overview. British Journal of Dermatology.

[ref18] (2007). Malassezia globosa and restricta: breakthrough
understanding of the etiology and treatment of dandruff and
seborrheic dermatitis through whole-genome analysis. Journal
of Investigative Dermatology Symposium Proceedings.

[ref19] (2007). Isolation and expression of a Malassezia
globosa lipase gene, LIP1. Journal of Investigative Dermatology.

[ref20] (2001). Dandruff
has an altered stratum corneum ultrastructure that is improved
with zinc pyrithione shampoo. Journal of the American
Academy of Dermatology.

[ref21] (2001). Seborrhoeic dermatitis and Pityrosporum (Malassezia)
folliculitis: characterization of inflammatory cells and
mediators in the skin by immunohistochemistry. British
Journal of Dermatology.

[ref22] (2011). Seborrheic dermatitis. Anais Brasileiros de Dermatologia.

[ref23] (1991). An immunological study in patients
with seborrhoeic dermatitis. Clinical and Experimental Dermatology.

[ref24] (2015). Network analysis identifies mitochondrial
regulation of epidermal differentiation by MPZL3 and FDXR. Developmental Cell.

[ref25] (2018). The genetic basis of seborrhoeic dermatitis: a review. Journal of the European Academy of Dermatology and Venereology.

[ref26] (2018). The role of zinc in the treatment of acne: a review of the
literature. Dermatologic Therapy.

[ref27] (2012). Zinc and skin: a brief summary. Dermatology Online Journal.

[ref28] (2007). Acrodermatitis enteropathica and an overview of zinc
metabolism. Journal of the American Academy of Dermatology.

[ref29] (2014). Hair zinc levels and
the efficacy of oral zinc supplementation in patients with atopic
dermatitis. Acta Dermato-Venereologica.

[ref30] (2014). Evaluation of the serum zinc level in erosive and nonerosive
oral lichen planus. Journal of Dentistry.

[ref31] (2002). Trace elements and antioxidant enzymes in Behcet’s disease. Rheumatology International.

[ref32] (2011). Serum zinc and copper status in Iranian
patients with pemphigus vulgaris. International Journal of
Dermatology.

[ref33] (1993). Analyses of serum copper
and zinc levels and copper/zinc ratios in skin diseases. Journal
of Dermatology.

[ref34] (2004). Vitamin and trace metal levels in recessive dystrophic
epidermolysis bullosa. Journal of the European Academy of Dermatology and Venereology.

[ref35] (2018). Evaluation of the serum zinc level in adult patients with
melasma: is there a relationship with serum zinc deficiency and
melasma?. Journal of Cosmetic Dermatology.

[ref36] (2017). Zinc as a gatekeeper of
immune function. Nutrients.

[ref37] (2014). Correlation between the severity and type of acne
lesions with serum zinc levels in patients with acne vulgaris. BioMed Research International.

[ref38] (2012). The 5 alpha-reductase
isozyme family: a review of basic biology and their role in
human diseases. Advances in Urology.

[ref39] (2009). Immunomodulation by statins: mechanisms
and potential impact on autoimmune diseases. Archivum Immunologiae et Therapiae Experimentalis.

[ref40] (2014). Zinc therapy in dermatology: a review. Dermatology Research and Practice.

[ref41] (2006). Toll-like receptor-mediated regulation of zinc homeostasis
influences dendritic cell function. Nature Immunology.

[ref42] (2014). The role of zinc in acne and
prevention of resistance: have we missed the “base” effect?. International Journal of Dermatology.

[ref43] (1995). Cations
inhibit specifically type I 5 alpha-reductase found in human
skin. Journal of Investigative Dermatology.

[ref44] (1993). Effect of a topical
erythromycin-zinc formulation on sebum delivery. Evaluation
by combined photometric-multi-step samplings with Sebutape. Clinical and Experimental Dermatology.

